# Supplementary material for: Strength of immune selection in tumors varies with sex and age
Source: Nat Commun. 2020 Aug 17;11:4128. doi: 10.1038/s41467-020-17981-0 (PMC7431859; doi:10.1038/s41467-020-17981-0)
Supplement: Supplementary file 1 — Supplementary Information [file 41467_2020_17981_MOESM1_ESM.pdf]

Strength of Immune Selection in Tumors Varies with Sex and Age

Castro et al.

Supplementary Figure 1.

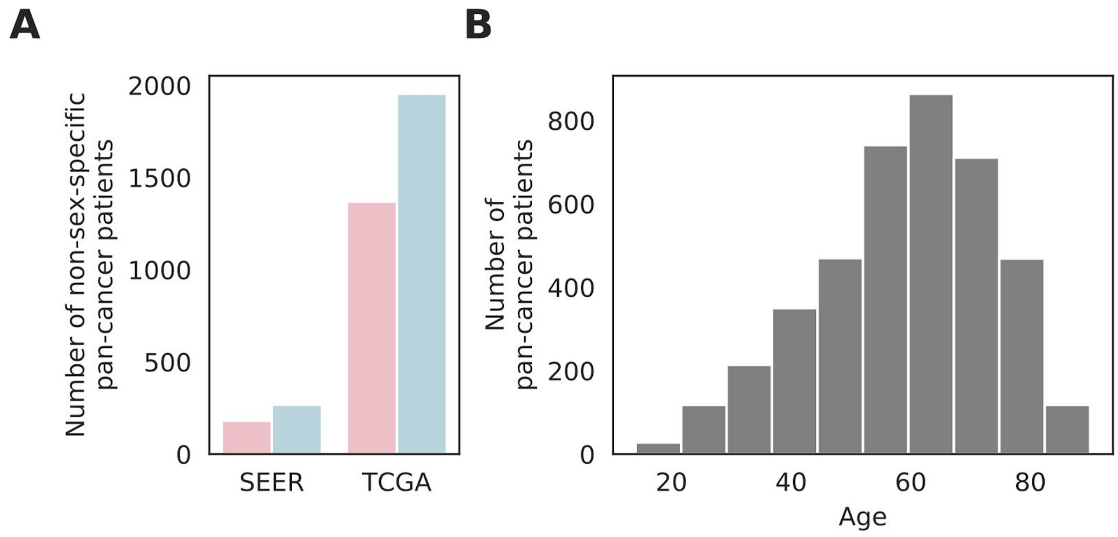

Overview of pan-cancer cohort. (A) A bar plot comparing the number of 3323 male and female patients in the pan-cancer cohort with sex-specific cancers (breast, cervical, ovarian, prostate, testicular, and uterine cancer) removed, compared to 450 new cases per 100,000 persons SEER non-sex-cancers cancer population statistics. (B) A histogram denoting the distribution of ages when patients were diagnosed with cancer for 4083 patients in the pan-cancer cohort. Sex-specific cancers were included for age analyses.

Supplementary Figure 2.

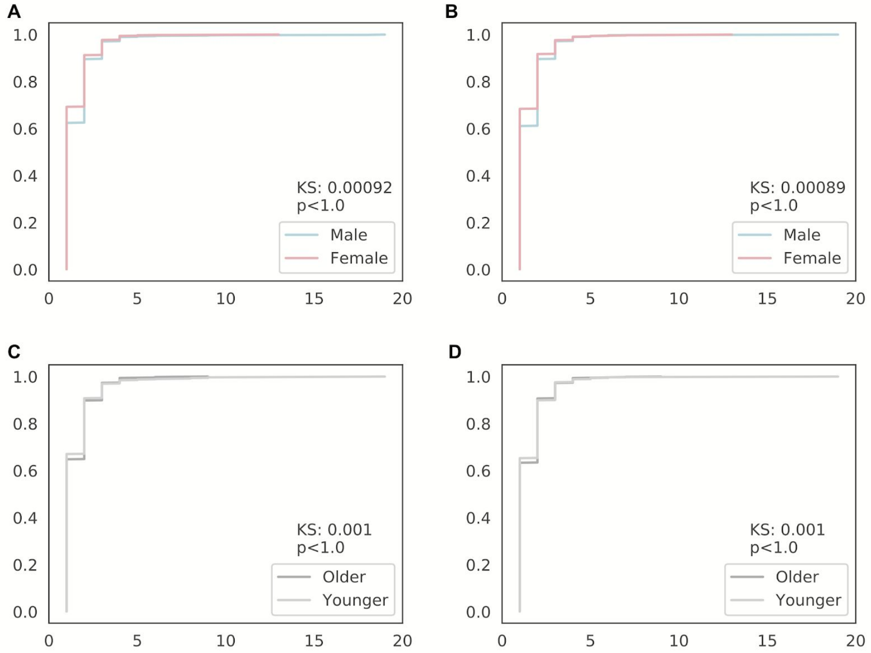

Driver mutation control analysis. The empirical cumulative distribution functions (ECDF) of driver mutations pan-cancer per cohort. Curves show the cumulative number of driver mutations in each cohort for microstatellite-stable patients with NetMHCpan-compatible calls. (A) ECDF for the MHC-I, sex-specific cohort, (B) ECDF for the MHC-II, sex-specific cohort, (C) ECDF for the MHC-I, age-specific cohort, and (D) ECDF for the MHC-II, age-specific cohort. P-values were obtained from the Kolmogorov-Smirnov two-sample test. Exact p-values all equal 1 for (A-D).

Supplementary Figure 3.

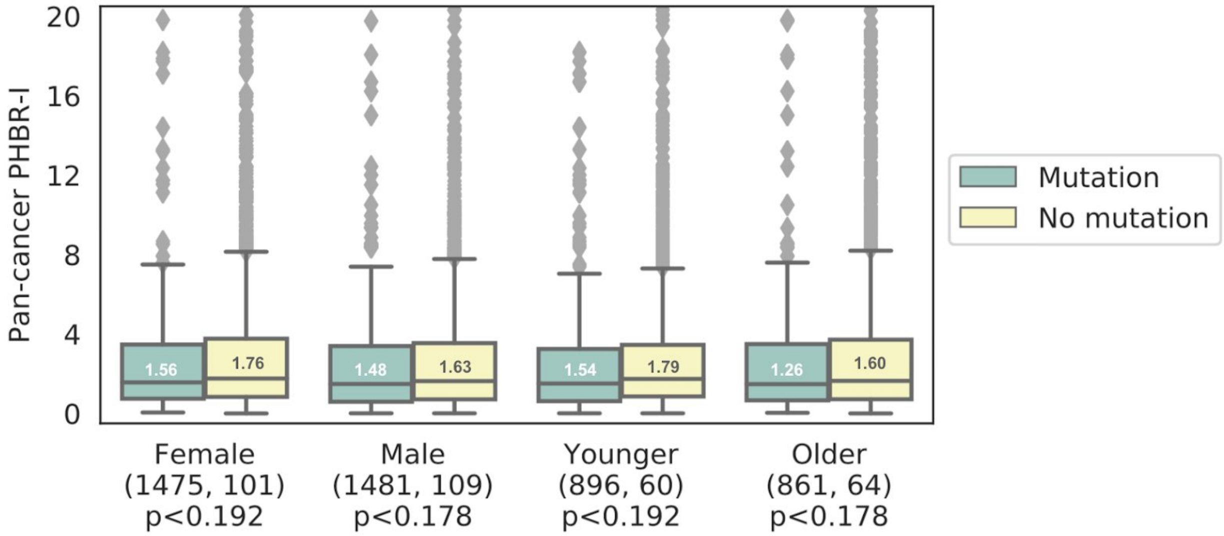

MHC-I mutation control analysis. Sex- and age-specific MHC presentation of common driver mutations for patients with and without MHC-I mutations. Box plots denote the distribution of PHBR-I scores for expressed driver mutations in female, male, younger, and older pan-cancer patients with and without MHC-I mutations. P-values were calculated using the one-tailed Mann Whitney U test. Exact p-values are: (A) 0.192, (B) 0.178, (C) 0.192, (D) 0.178. Median values are indicated in each boxplot. All boxplots include the median line, the box denotes the interquartile range (IQR), whiskers denote the rest of the data distribution and outliers are denoted by points greater than +/- 1.5 * IQR.

Supplementary Figure 4.

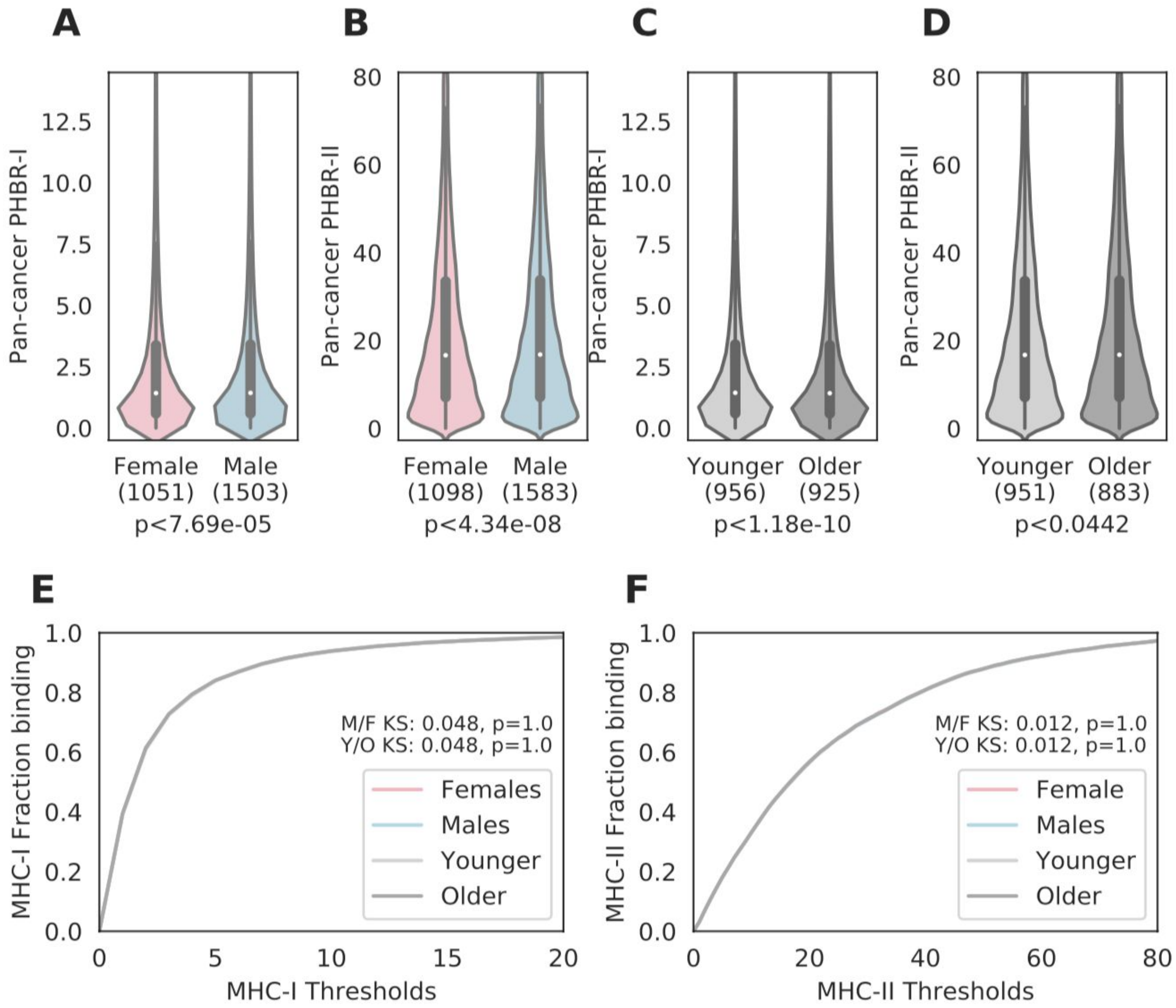

Sex- and age-specific MHC presentation of common driver mutations. (A-D) Violin plots denoting the sex and age stratified distribution of (A, C) PHBR-I and (B, D) PHBR-II scores across all common cancer driving mutations. P-values were calculated using the one-tailed Mann Whitney U test. Exact p-values are: (A) 7.69e-05, (B) 4.34e-08, (C) 1.18e-10, (D) 0.0442. Effect sizes were calculated using Cliff's d: (A) r=-0.00276, (B) r=-0.00381, (C) r=-0.00529, (D) r=-0.00144. Median PHBR scores are: (A) 1.42 F, 1.43 M, (B) 16.65 F, 16.81 M (C) 1.44 Y, 1.42 O (D) 16.72 Y, 16.75 O. (E, F) Empirical cumulative distribution functions showing the fraction of driver mutations predicted to bind to (E) MHC-I and (F) MHC-II at different PHBR score thresholds. Distributions were compared using the Kolmogorov-Smirnov two-sample test.

Supplementary Figure 5.

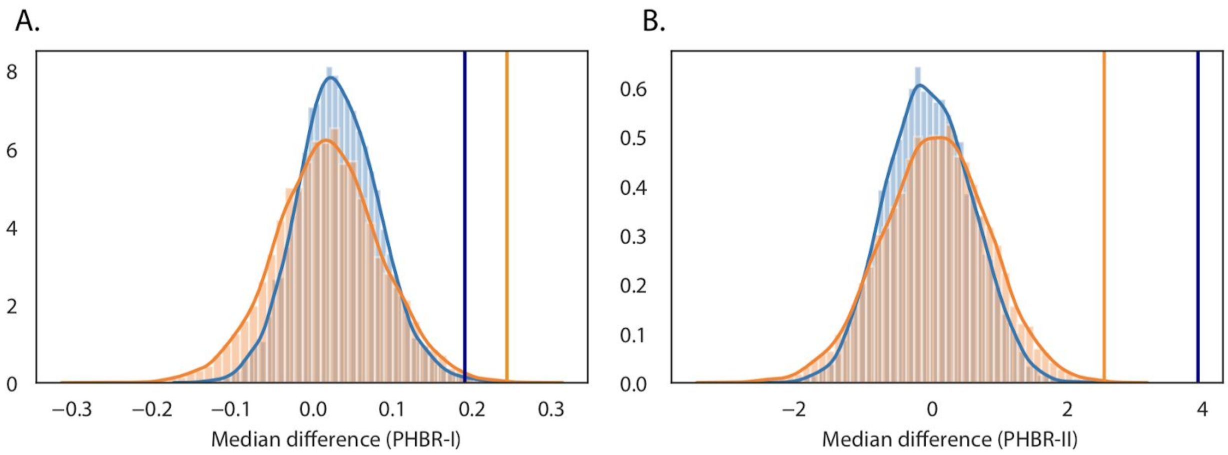

Shuffling driver mutations control analysis. Distributions of the median difference in (A) PHBR-I and (B) PHBR-II scores between sex-specific (blue) and age-specific (orange) patient groups estimated by shuffling driver mutations between patients 10,000 times while maintaining constant column and row counts. Solid lines indicate the observed score differences between cohorts.

Supplementary Figure 6.

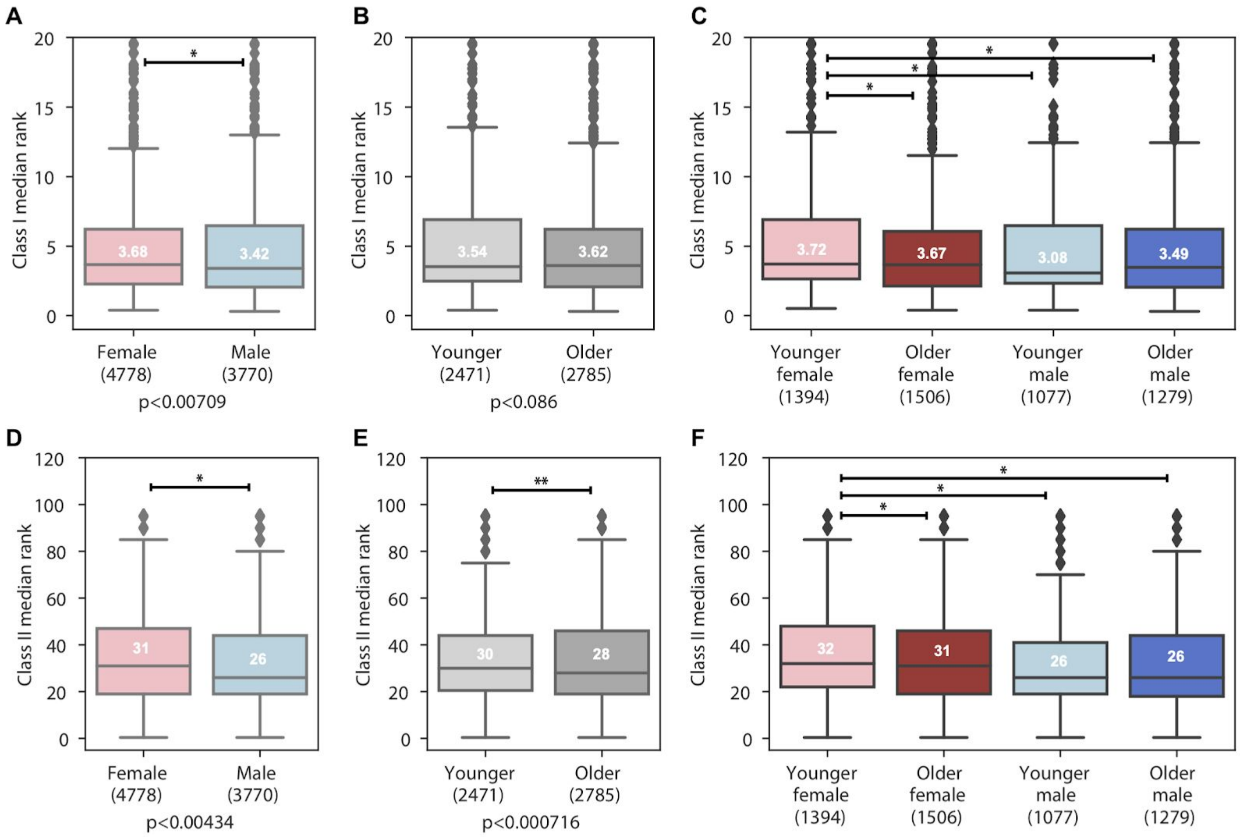

NetMHCpan alternate affinity analysis. Boxplots showing the distribution of median NetMHCpan (A-C) class I and (D-F) class II affinity scores for observed, expressed driver mutations across sex and age in our cohort. P-values were calculated using the one-tailed Mann Whitney U test. The Benjamini-Hochberg method was used to adjust for multiple comparisons for (C) and (F). Exact p-values are: (A) 0.00709, (B) 0.086, (C) YF, YM: 0.016; YF, OM: 0.019; YF, OF: 0.043; OF, YM: 0.27; OF, OM: 0.33; YM, OM: 0.42, (D) 0.00434, (E) 7.16e-04, (F) YF, OM: 4.64e-05; YF, YM: 0.0005; YF, OF: 0.0008; YM, OM: 0.24; OF, OM: 0.21; OF, YM: 0.43. Y=younger, O=older, F=female, M=male. One asterisk indicates p-values <0.05 and two asterisks indicates p-values <0.001. Median values are indicated in each boxplot. All boxplots include the median line, the box denotes the interquartile range (IQR), whiskers denote the rest of the data distribution and outliers are denoted by points greater than +/- 1.5 * IQR.

Supplementary Figure 7.

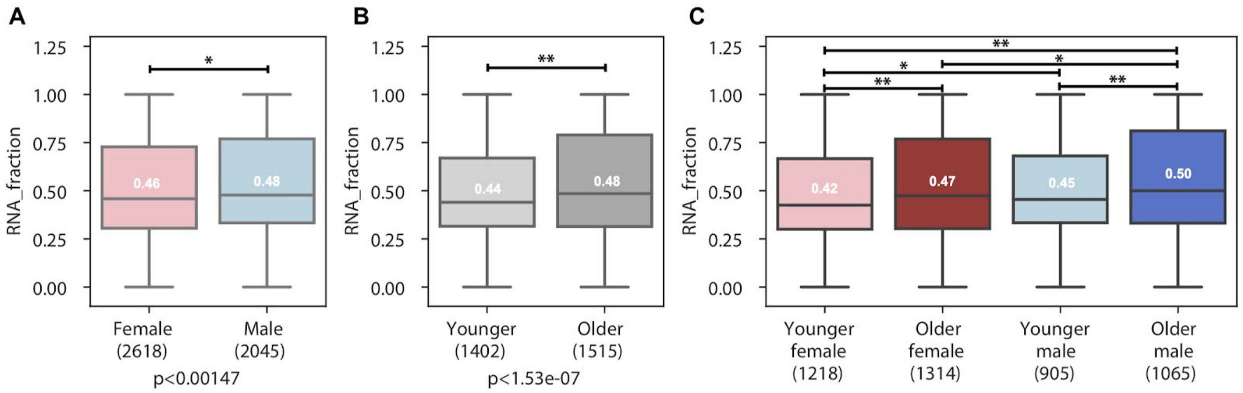

Sex- and age-specific analysis of mutation RNA fraction. Box plots showing the distribution of fraction of RNA reads supporting the mutated allele in (A) female and male patients, (B) younger and older patients, and (C) integrated sex- and age-specific patient cohorts. P-values were calculated using the one-tailed Mann Whitney U test. The Benjamini-Hochberg method was used to adjust for multiple comparisons for (C). Exact p-values are: (A) 0.000147, (B) 1,53e-07, (C) YF, OM: 1.19e-08; YF, OF: 0.0003; YM, OM: 0.0006; YF, YM: 0.008; OF, OM: 0.015; OF, YM: 0.11. Y=younger, O=older, F=female, M=male. One asterisk indicates p-values <0.05 and two asterisks indicates p-values <0.001. Median values are indicated in each boxplot. All boxplots include the median line, the box denotes the interquartile range (IQR), whiskers denote the rest of the data distribution and outliers are denoted by points greater than +/- 1.5 * IQR.

Supplementary Figure 8.

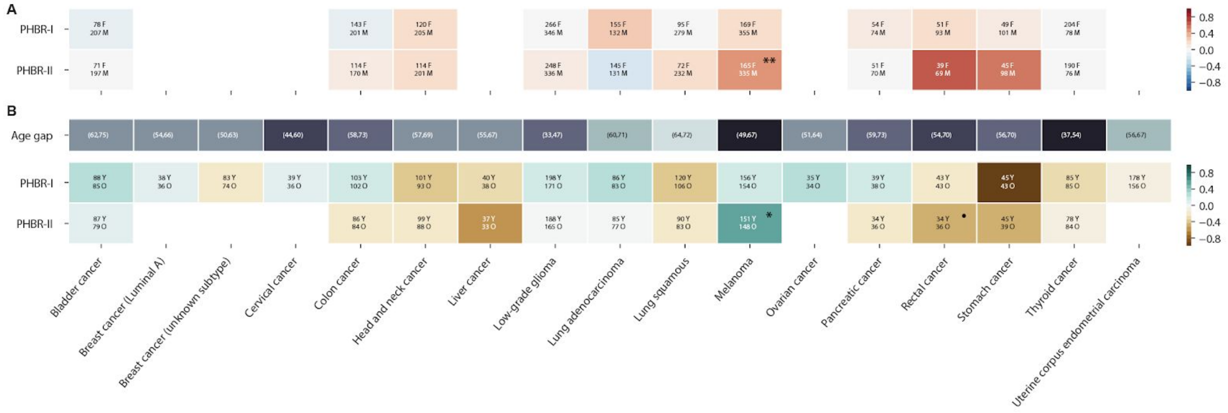

Disease-specific sex- and age-specific analysis of driver affinities. Heatmaps showing disease-specific PHBR-I and PHBR-II median ratios for (A) females vs. males where red coloring indicates higher median female PHBR distributions, and blue coloring indicates higher median male PHBR distributions. (B) Younger vs. older patients where green coloring indicates higher median younger PHBR distributions and yellow indicates higher median older PHBR distributions. "Age gap" shows disease-specific age thresholds (30th and 70th percentile), with darker coloring indicating a wider age gap and vice versa. A minimum of 30 patients in each group was required; blanks indicate that fewer than 30 samples were available in one of the categories. P-values were calculated using the one-tailed Mann Whitney U test. The Benjamini-Hochberg method was used to adjust for multiple comparisons for (A) and (B). Exact p-values for significant comparisons are: (A) 0.00098 for PHBR-II in melanoma, (B) 0.04 for PHBR-II in melanoma, and 0.07 for PHBR-II in rectal cancer. One asterisk indicates p-values <0.05, two asterisks indicates p-values <0.001, and a dot indicates p-values <0.1.

Supplementary Figure 9.

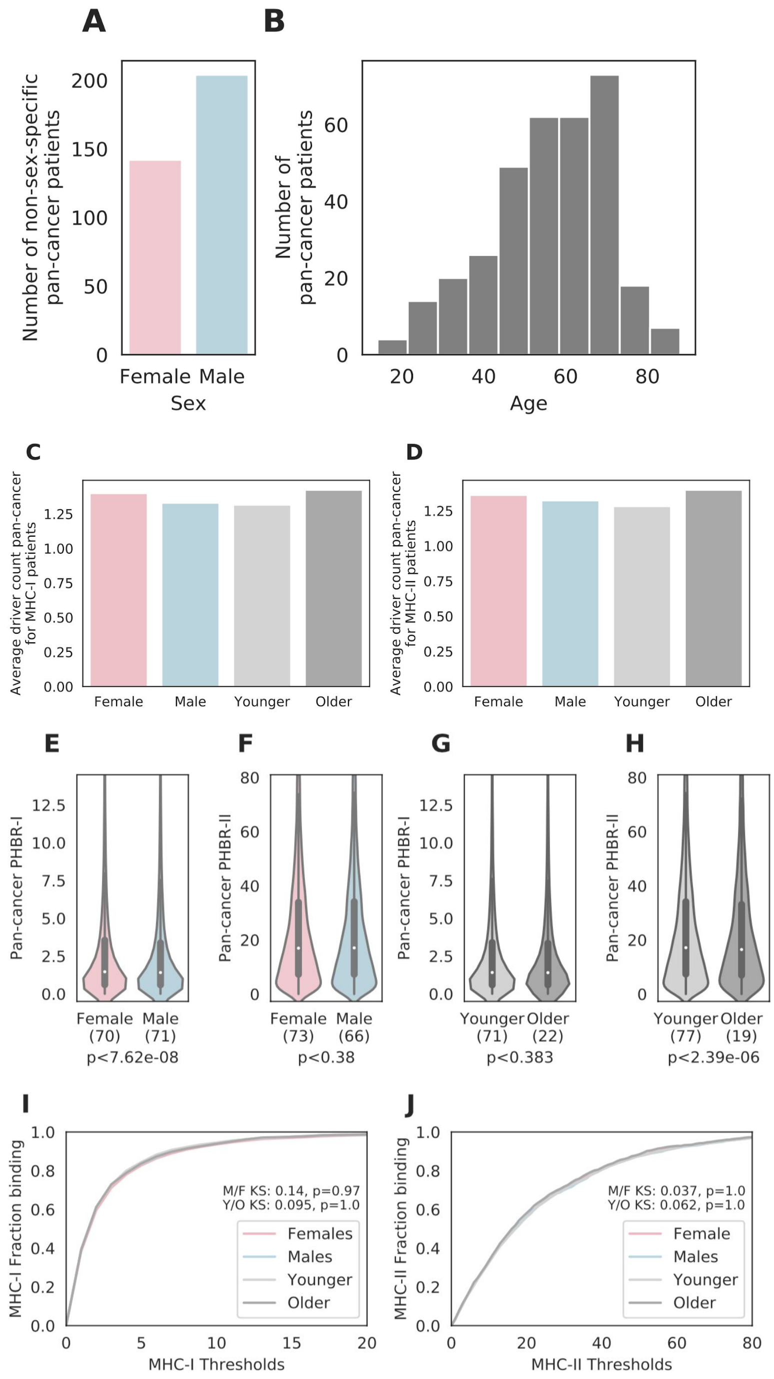

Overview of the validation cohort. (A) A bar plot comparing 346 male and female patients in the pan-cancer validation cohort. (B) A histogram denoting the distribution of ages when patients were diagnosed with cancer for 335 patients in the pan-cancer validation cohort. (C-D) Bar plots denoting the average number of driver mutations for 346 patients in each sex- and age-specific cohort for (C) patients with MHC-I calls, and (D) patients with MHC-II calls. Median PHBR scores are: (A) 1.46 F, 1.41 M, (B) 16.97 F, 17.07 M (C) 1.41 Y, 1.40 O (D) 17.15 Y, 16.50 O. (E-H) Violin plots denoting the distribution of

(E, G) PHBR-I and (F, H) PHBR-II scores across all common cancer driving mutations. P-values were calculated using the one-tailed Mann Whitney U test. Exact p-values are: (E) 7.62e-08, (F) 0.38, (G) 0.383, (H) 2.39e-06. Effect sizes were calculated using Cliff's d: (A) r=-0.016, (B) r=-0.000938, (C) r=-0.00132, (D) r=-0.0212. (I, J) Empirical cumulative distribution functions showing the fraction of driver mutations predicted to bind to (I) MHC-I and (J) MHC-II at different PHBR score thresholds. Distributions were compared using the Kolmogorov-Smirnov two-sample test.

Supplementary Table 1.

	Parametric coefficients	Estimate	Pr(>|z|)
PHBR-I analysis	**PHBR-I**	**0.13**	**7.54e-22**
	Sex	-0.003	0.93
	**Age**	**-0.0026**	**0.02**
	**PHBR-I:Sex**	**0.08**	**0.003**
	**PHBR-I:Age**	**-0.0028**	**0.001**
PHBR-II analysis	**PHBR-II**	**0.33**	**2.05e-72**
	Sex	-0.05	0.22
	Age	-0.002	0.17
	**PHBR-II:Sex**	**0.16**	**2.73e-05**
	**PHBR-II:Age**	**-0.004**	**0.000518**

Quantitative estimate of the association between PHBR score and mutation occurrence in sex- and age-specific TCGA cohorts. Estimates and p-values are shown for a generalized additive model with random effects relating PHBR-I and PHBR-II scores to the occurrence of driver mutations observed ≥2 times in the TCGA cohort. P-values were calculated via Wald tests using the Bayesian covariance matrix for the coefficients. Variables and their respective estimates and p-values have been bolded if significant ($p<0.05$).

Supplementary Table 2.

	Parametric coefficients	Estimate	Pr(>|z|)
Sex analysis	**PHBR-I**	**0.06**	**0.002**
	**PHBR-II**	**0.33**	**1.9e-41**
	Sex	-0.03	0.54
	PHBR-I:Sex	0.075	0.07
	**PHBR-II:Sex**	**0.12**	**0.01**
Age analysis	**PHBR-I**	**0.06**	**0.004**
	**PHBR-II**	**0.33**	**2.2e-40**
	Age	-0.0021	0.17
	**PHBR-I:Age**	**-0.0044**	**0.0005**
	**PHBR-II:Age**	**-0.004**	**0.01**

Quantitative estimate of the association between PHBR score and mutation occurrence in sex- and age-specific TCGA cohorts, without tumor types significantly associated with sex-specific mutational signature ratios. Estimates and p-values are shown for a generalized additive model with random effects relating PHBR scores to occurence of driver mutations observed ≥2 times in the TCGA cohort. P-values were calculated via Wald tests using the Bayesian covariance matrix for the coefficients. Variables and their respective estimates and p-values have been bolded if significant ($p<0.05$).

Supplementary Table 3.

	Parametric coefficients	Estimate	Pr(>|z|)
Sex analysis	PHBR-I	-0.17303	0.0576
	**PHBR-II**	**0.26725**	**0.0464**
	Sex	0.21348	0.2901
	PHBR-I:Sex	-0.09819	0.5917
	PHBR-II:Sex	0.30335	0.2647
Age analysis	PHBR-I	-0.153878	0.087
	**PHBR-II**	**0.287683**	**0.0302**
	Age	-0.009375	0.1771
	PHBR-I:Age	-0.007862	0.2371
	PHBR-II:Age	-0.009064	0.3329

Quantitative estimate of the association between PHBR score and mutation occurrence in sex- and age-specific validation cohorts. Estimates and p-values are shown for a generalized additive model with random effects relating PHBR scores to occurence of driver mutations observed in the validation cohort. P-values were calculated via Wald tests using the Bayesian covariance matrix for the coefficients. Variables and their respective estimates and p-values have been bolded if significant ($p<0.05$).
