# Supplementary material for: Strength of immune selection in tumors varies with sex and age
Source: Nat Commun. 2020 Aug 17;11:4128. doi: 10.1038/s41467-020-17981-0 (PMC7431859; doi:10.1038/s41467-020-17981-0)
Supplement: Supplementary file 3 — Description of Additional Supplementary Files [file 41467_2020_17981_MOESM3_ESM.pdf]

## Description of Additional Supplementary Files

### Supplementary Dataset 1

**Description:** Summary of validation patients. Columns describe patient IDs, tumor type, age of diagnosis and sex.”
